# Editorial: Immune-genetic dynamics in disease progression and therapeutic strategies

**DOI:** 10.3389/fimmu.2026.1829346

**Published:** 2026-03-23

**Authors:** Zhongbao Zhou, Zhuoqi Cheng, Yong Zhang

**Affiliations:** 1 Department of Urology, Beijing TianTan Hospital, Capital Medical University, Beijing, China; 2 Department of Internal Medicine 3, Rheumatology and Immunology, Friedrich-Alexander-Universität Erlangen-Nürnberg (FAU) and Universitätsklinikum Erlangen, Erlangen, Germany

**Keywords:** biomarkers, disease progression, genetic modifications, immune microcosms, personalized medicine

## Introduction

The immune microenvironment and genetic alterations are central to understanding disease progression and therapeutic responses (1). Research has established that complex interactions within the immune microenvironment play pivotal roles in maintaining tissue homeostasis and therapeutic efficacy. Advances in elucidating how immune cells and genetic factors interact have significant implications for disease diagnosis, progression, and management-particularly in inflammatory diseases, neurologic disorders, and cancers. Emerging evidence suggests that dysregulation of immune microenvironments is directly linked to pathological states and offers promising potential for developing novel therapeutic approaches (2). This Research Topic explores the intricate interplay between genetic modifications and immune microenvironments and their collective impact on disease progression and treatment response. Our objective is to elucidate how genetic changes influence immune function and contribute to disease development or suppression. By examining patterns of gene regulation and their effects on the immune landscape, we seek to advance personalized medicine approaches with tailored diagnostic and therapeutic strategies based on individual genetic and immune profiles.

## Publication summary

We have carefully selected seven high-quality publications for this Research Topic. Six studies investigated the role of the immune microenvironment in identifying molecular markers across different tumor types, while one review examined recent advances in PD-1/PD-L1 inhibitor-based combination therapy for cancer treatment. Below is a summary of these important contributions:

## Study 1: therapeutic potential of Huoshan dendrobium Zengye Jiedu formula for radiation-induced oral mucositis

Radiation-induced oral mucositis (RIOM) causes mucosal ulceration, pain, and dysphagia, disrupting cancer treatment and diminishing patients’ quality of life. Its pathogenesis involves inflammatory imbalance, immune dysregulation, microbial invasion, and cytokine storms, yet current therapeutic approaches remain inadequate. Liu et al. investigated the therapeutic potential and mechanism of Huoshan Dendrobium Zengye Jiedu Formula (HDZJF) for RIOM treatment. Their findings demonstrate that HDZJF comprises 102 bioactive components targeting 379 RIOM-related proteins, with functional enrichment analysis indicating effects on angiogenesis, inflammation, and apoptosis via the PI3K-AKT signaling pathway. Molecular docking revealed strong HDZJF–target affinities; *in vivo* studies showed that HDZJF reduced inflammation, promoted mucosal healing, improved body weight maintenance, and modulated EGFR/PI3K/AKT signaling. These findings support HDZJF’s translational potential for RIOM treatment and warrant further clinical evaluation.

## Study 2: isoform-specific roles of neuropilin in tumor-associated macrophages and tumor microenvironment remodeling

Neuropilin (NRP) isoforms NRP1 and NRP2 play distinct, cancer-type-specific roles in shaping the tumor microenvironment (TME) through tumor-associated macrophages (TAMs). Han et al. analyzed NRP isoform expression patterns in TAMs and their associated transcriptomic programs using single-cell RNA sequencing across two cancer models: clear cell renal cell carcinoma (ccRCC) and skin cutaneous melanoma (SKCM). The analysis identified selective upregulation of NRP1 in ccRCC TAMs and NRP2 in SKCM TAMs. Both NRP1+ and NRP2+ macrophages exhibited M2-like polarization characteristics associated with immune suppression and extracellular matrix degradation. Functional enrichment analysis revealed that NRP1-associated gene sets enrich for angiogenesis and VEGF signaling pathways, while NRP2 demonstrates dual, context-dependent TME functions. Collectively, these data reveal isoform-specific TAM mechanisms and support the rationale for isoform-targeted immunomodulation strategies in cancer treatment.

## Study 3: PRSS22 as a potential immunotherapeutic target in colorectal cancer

Colorectal cancer (CRC) exhibits high heterogeneity with complex pathogenesis, posing significant challenges for prognosis and treatment. Advances in single-cell RNA sequencing enable deep exploration of tumor microenvironment dynamics and cellular interactions. Xu et al. employed single-cell transcriptomic analysis to identify a tumor cell subcluster exhibiting high PRSS22 expression that demonstrated enhanced proliferation and migration capabilities and correlated with immune dysregulation. Functional studies demonstrated that PRSS22 downregulation reduced CRC cell proliferation, migration, and invasion capacity, highlighting PRSS22 as a potential immunotherapeutic target for CRC management.

## Study 4: genotype-guided dosing of calcineurin inhibitors to improve hematopoietic stem cell transplantation outcomes

Calcineurin inhibitors (CNIs) such as cyclosporine A (CsA) are widely used to prevent graft-versus-host disease (GVHD) following hematopoietic stem cell transplantation (HSCT); however, therapeutic responses vary considerably due to transporter and metabolic single nucleotide polymorphisms (SNPs). Ji et al. genotyped CsA-related SNPs in 128 pediatric patients receiving CsA-based GVHD prophylaxis and examined their associations with post-transplant complications and survival outcomes. The results demonstrated that CsA-related SNPs significantly influence complication risk and survival, supporting the clinical utility of genotype-guided dosing strategies to improve HSCT outcomes and reduce adverse events.

## Study 5: macrophage polarization-related gene signature as a biomarker in rectal cancer

Macrophage polarization plays a critical role in shaping the tumor microenvironment and drives rectal cancer progression; however, the metabolic regulators underlying these processes remain poorly defined. Xu et al. constructed a macrophage polarization gene signature (MPGS) by integrating weighted gene coexpression network analysis (WGCNA) with machine learning algorithms in two cohorts (GSE87211, n=363; TCGA, n=177), and investigated its prognostic value. Mechanistic exploration via single-cell RNA sequencing, immunohistochemistry, and *in vitro* functional assays identified PYGM as a key metabolic and immunological regulator. These findings indicate that MPGS and PYGM are potential biomarkers for risk stratification and treatment guidance in rectal adenocarcinoma and may improve the overall management of colorectal cancer.

## Study 6: PLEK as an immune-metabolic prognostic regulator in osteosarcoma

osteosarcoma (OS) is a highly metastatic malignancy in which immune-metabolic reprogramming drives disease progression; however, the molecular mechanisms linking these processes remain elusive. Zou et al. identified immune-metabolic biomarkers through integrated bioinformatic analysis, identifying pleckstrin (PLEK) as a central hub. Their analysis revealed that PLEK upregulation predicted improved survival outcomes and enhanced immune infiltration. Single-cell transcriptomic analysis showed PLEK was enriched in macrophage clusters exhibiting active glycolysis and oxidative phosphorylation. Functional studies demonstrated that PLEK knockdown promoted OS cell proliferation and invasion. These results establish PLEK as a prognostic immune-metabolic regulator and a promising therapeutic target in osteosarcoma.

## Study 7: PD-1/PD-L1 inhibitor-based combination strategies in cancer therapy

Immunotherapy, particularly PD-1/PD-L1 checkpoint inhibitors, represents a promising therapeutic strategy by enhancing anti-tumor immune recognition while minimizing damage to normal tissues. Tong et al. comprehensively reviewed recent evidence on PD-1/PD-L1 inhibitors used in combination with chemotherapy, other immune checkpoint inhibitors (ICIs), or traditional Chinese medicine (TCM). The review aims to clarify synergistic mechanisms underlying these combinations, discuss clinical applications and efficacy data, and highlight TCM’s potential in informing future anti-tumor therapeutic strategies and clinical practice guidelines.

## Conclusion

This Research Topic brings together diverse perspectives on the interplay between genetic factors and immune microenvironments in disease pathogenesis and therapeutic response. The selected publications collectively advance our understanding of personalized medicine approaches and provide a foundation for developing more effective, individualized treatment strategies across multiple disease contexts.
